# A factorization method for the classification of infrared spectra

**DOI:** 10.1186/1471-2105-11-561

**Published:** 2010-11-15

**Authors:** Carsten Henneges, Pavel Laskov, Endang Darmawan, Jürgen Backhaus, Bernd Kammerer, Andreas Zell

**Affiliations:** 1 Zentrum für Bioinformatik Tübingen, Eberhard Karls Universität Tübingen, Sand 13, Tübingen, Germany; 2 Institut für Instrumentelle Analytik und Bioanalytik, Hochschule Mannheim, Paul-Wittsack-Straße 10, Mannheim, Germany; 3 Zentrum für Biosystemanalyse ZBSA, Albert-Ludwigs-Universität Freiburg, Habsburgerstraße 49, Freiburg, Germany

## Abstract

**Background:**

Bioinformatics data analysis often deals with additive mixtures of signals for which only class labels are known. Then, the overall goal is to estimate class related signals for data mining purposes. A convenient application is metabolic monitoring of patients using infrared spectroscopy. Within an infrared spectrum each single compound contributes quantitatively to the measurement.

**Results:**

In this work, we propose a novel factorization technique for additive signal factorization that allows learning from classified samples. We define a composed loss function for this task and analytically derive a closed form equation such that training a model reduces to searching for an optimal threshold vector. Our experiments, carried out on synthetic and clinical data, show a sensitivity of up to 0.958 and specificity of up to 0.841 for a 15-class problem of disease classification. Using class and regression information in parallel, our algorithm outperforms linear SVM for training cases having many classes and few data.

**Conclusions:**

The presented factorization method provides a simple and generative model and, therefore, represents a first step towards predictive factorization methods.

## Background

Bioinformatics data analysis often deals with additive mixtures of signals from unknown interfering sources. In the majority of cases, only class labels are known for each sample, which hampers the estimation of the original source signals. An example of such a situation is the search for metabolic features in blood within different patient groups. In blood, several signal sources add up as each single organ may submit hormones contributing its state into this complex mixture. For instance, adipocytes secrete the hormone leptin to indicate their state. This signal is then recognized in the hypothalamus to regulate the appetite. At the same time, insulin is secreted by pancreatic beta cells for the regulation of the blood sugar. Both peptide hormones are present within the blood while their regulation results in different outcomes. However, both signals are also hidden within a huge and noisy background of further signals also present in the blood stream. Consequently, a large number of samples must be taken to clearly identify an unknown signal. Infrared (IR) spectroscopy is a rapid method for detecting signals in biological samples. It relies on quantities of 1 μ*l *size that can be easily obtained and it is fast: measuring a complete sample where each single molecule is detected requires a total time of 30 s on a Bruker Tensor 37.

**Figure 1:**
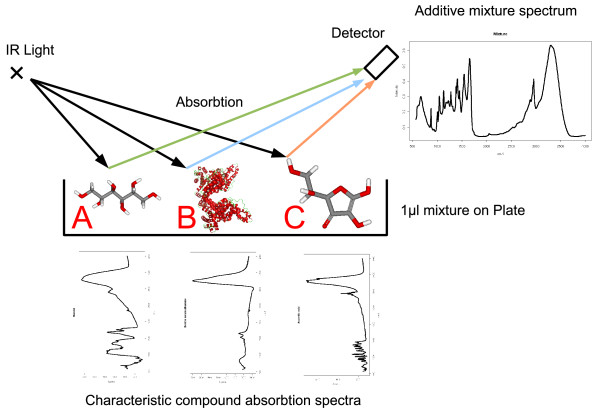
**The principle of IR spectroscopy**. For IR spectroscopy light is cast on a sample of compounds. Depending on its constitution, each compound has a characteristic absorption pattern for the received light. This results in an additive superposition of absorption, which is subsequently recorded as a mixture spectrum. Here, each compound contributes its absorption fingerprint quantitatively to the mixture. The fingerprint can be used for compound identification, because the absorption depends on all paths through the compound that oscillate such that the permanent dipole changes.

The principles of IR spectroscopy, see for instance [1], are illustrated in Figure 1. IR spectroscopy can be used for the quantification of known compounds or for structural elucidation of unknown molecules. An IR source emits light towards a sample solution of chemical compounds. IR radiation is absorbed by chemical compounds as motion energy when the absorbed energy fulfills the resonance condition of a tone or related overtones. In this way, IR spectroscopy detects oscillations of bonds. As an additional condition, IR spectroscopy requires that oscillations lead to a periodical change of the molecular dipole moment. Consequently, compounds having no dipole are IR inactive. However, in the case of an IR active compound functional groups can be identified by their characteristic absorption bands, and thus give hints for structural elucidation. Alternatively, compounds can be identified through their characteristic fingerprint region within their IR spectra. This unique characteristic absorption fingerprint completely depends on the molecular constitution, because each path through a compound that is associated with a change in dipole moment absorbs at a characteristic wave length. All such paths of various lengths yield the characteristic absorption spectrum and uniquely identify the compound. Thus, the prediction of the IR spectrum of a compound is a hard task. Finally, we want to note that the IR detector records a mixture signal of *all *compounds present in the sample. Consequently, each single molecule present in the 1 *μ*l sample contributes to the signal, whether it is known or not. Then, the vibration spectrum represents a complex " fingerprint" of the biochemical condition of the sample wherein single compounds are not recognized any more.

However, all diseased changes are included in detail integrately such that the sample can be analyzed objectively and without knowing disease markers with the IR-spectroscopy. In this way, IR spectroscopy has a great potential as a method for early diagnosis and therapy control [2-4]. Analyzing IR spectra is however a complex signal processing problem.

Nonetheless, there exist algorithms that are able to separate additive signals into estimated subcomponents. Examples for these methods are Non-negative Matrix Factorization (NMF) [5] or Independent Component Analysis (ICA) [6,7]. Both compute a generative additive signal model that is fitted to data samples to estimate the basic subsignals each data sample is composed of. However, IR spectra do not completely fulfill sparseness or smoothness constraints used by ICA or NMF completely, see [8]. Moreover, these methods are not designed for training on data with classification labels nor do they yield predictive models. In this work, we solve the class assignment problem and design a factorization method using a generative additive model that can be trained on data samples having class labels. For each class label, a factor signal is computed that, when exceeding a learned threshold, predicts the specific label. Therefore, our method can be trained on cheap IR spectra using class information and extract meaningful components from these signals, which leads to further insight into data and a predictive model.

## Methods

This section develops the new predictive matrix factorization algorithm named *BrierScoreMF *for IR spectra. First, we motivate and define the problem. Then, we introduce factorization and classification loss functions and their matrix formulations. Finally, we derive the *BrierScoreMF *algorithm.

### 1.1 Problem formulation

In daily practice, bioinformatics often deals with signals from interfering sources. Each source could have considerable impact on the final interpretation of the signal. For instance, consider endocrine signaling. The endocrine system is composed of glands secreting a hormone into the blood stream. Within certain ranges, these signals represent the normal body state. However, increased signals may indicate a disease state, e.g. oncogenesis [9]. Thus, measuring all endocrine signals yields a superposition of healthy and disease signal combinations that have to be separated to diagnose the physical state. Moreover, disease signals may be combinations of coregulated signals not originating from a single signal source. In practice, measured signals are only grouped by disease classes raising the question for the characteristic shape of the disease signals.

Thus, we are dealing with two simultaneous problems: A signal decomposition problem and a classification problem that is based on the signal decomposition. A practical approach would try to learn the signals from given data samples.

Matrix factorization methods are convenient algorithms for the signal decomposition task [5]. These methods solve the problem of finding the decomposition *X *= *AS *for any matrix *X*. In general, this problem is ill-posed. However, using constraints restricts the number of feasible solutions, which can be found by local optimization algorithms. Commonly used restrictions comprise constraints for the statistical independence of signals [6] as well as non-negativity or sparsity of coefficients in *A *[5]. Up to now no factorization method is known using class labels, therefore our approach includes constraints for classification that are needed to learn from IR spectra obtained in clinical studies.

We begin with developing our predictive factorization algorithm. Given *n *pairs $\left({\vec{x}}_{i},y_{i}\right)$ of data samples comprising signals ${\vec{x}}_{i}\in {\mathbb{R}}^{d}$ and *k *classes *y_i _*∈ *C *= {*c _i_*, . . .,*c _k_*}, we define the following matrices

(1) $$\text{Design matrix}\;X={\left({\vec{x}}_{i}\right)}^{T}\forall i$$

(2) $$\text{Class matrix}\;Y=\left(y_{ij}\right)\forall i\;\text{where}y_{ij}=\left\{ \begin{array}{l} +1,\text{ if }x_{i}\text{ has class }c_{j}\\ -1,\;else \end{array}\right..$$

The dimensions are *X *∈ ℝ^*n *× *d *^and *Y *∈ ℝ*^n × k^*. Thus, each row in *X *defines a measured signal and relates to a row in *Y *containing binary class information.

Searching for a factorization into *signals *${\vec{s}}_{j}\in {\mathbb{R}}^{d}$ and *coefficients a_ij _*∈ ℝ, we want that

(3) $${\vec{x}}_{i}=\sum_{j=0}^{k}a_{ij}{\vec{s}}_{j}\forall i\Leftrightarrow X=AS$$.

where *A *∈ ℝ*^n × k ^*and *S *∈ ℝ*^k × d^*. Equation (3) means that each signal is a linear combination of *k *different source signals and defines the general factorization problem in matrix formulation with respect to *A *and *S*. In practice, noise hampers the inference of the ${\vec{s}}_{j}$ and, consequently, this condition is not fulfilled exactly by any solution. However, we will see that in our special case the problem only reduces to finding a suitable *S*. For classification, we propose a linear approach using a threshold. Therefore, we want that

(4) $$y_{ij}=\text{signum}\;\left(a_{ij}-b_{j}\right)\forall i,j$$

where $\vec{b}\in {\mathbb{R}}^{k}$ is a column threshold vector of the factorization. If the signal fraction exceeds a certain threshold, this will indicate the class membership within our prediction model.

### 1.2 Factorization loss functions

In general, factorization algorithms focus on the signal side of the problem. These methods optimize special distance functions between probability distributions, referred to as divergences, to estimate *A *and *S*. It can be shown that optimizing *A *and *S *in parallel is a non-convex optimization problem. Commonly used divergences include the Frobenius norm as well as the Kullback-Leibler divergence. Other exemplary divergences are the Itakura-Saito divergence and the families of *α*- and *β*-divergences [5].

However, in this work we will rely on the Frobenius norm between *X *and *AS *for divergence. Thus, we define the reconstruction error part of our loss function as

(5) $$ℱ\left(X,AS\right)={\left\|X-AS\right\|}_{F}$$

where

$${\left\|Z\right\|}_{F}\equiv \sqrt{\sum_{i,j}z_{i,j}^{2}}\Leftrightarrow {\left\|Z\right\|}_{F}^{2}=\text{tr}Z^{T}Z$$

for some matrix *Z*. Here, tr denotes the trace of a matrix.

We have chosen the Frobenius norm as divergence for the reconstruction error, because it easily allows to compute the matrix differentials of an expression. This will simplify the search for possible solutions in section 1.4.

### 1.3 Classification loss functions

Classification algorithms focus on the inference of a predictive model for a target variable from training data. Therefore, they optimize classification loss functions that penalize false predictions to find the most probable parametrization of a model. Convenient loss functions comprise the Brier Score [10], the SVM loss [11], the logistic loss [12], as well as the Misclassification loss function.

We chose the Brier-Score [10] as it also can be expressed in terms of matrix computations. Let *y *∈ {-1, +1} be the class label and let *E*[.] denote the expectation operator. Then the Brier-Score is defined as

(6) $$E\left[{\left(yf\left(x\right)-1\right)}^{2}\right]$$

where *f*(*x*) is a parametrized model function.

Now, consider equation (4) and define the matrix *V *to contain the signum arguments

$$V\equiv {\left(a_{ij}-b_{j}\right)}_{n\times k}=A-1_{n\times 1}{\vec{b}}^{T}$$

where $\vec{b}\in {\mathbb{R}}^{k\times 1}$ is the vector of column thresholds. Then, the Brier-Score can be written as a matrix function from ${\mathbb{R}}^{n\times k}\mapsto \mathbb{R}$ as

$$ℬ\left(Y,V\right)=\kappa \text{tr}{\left(Y\circ V-1_{n\times k}\right)}^{T}\left(Y\circ V-1_{n\times k}\right)$$

where $\kappa =\frac{1}{nk}$,*Y *is the class matrix, **1***_n×k _*an *n×k *matrix of ones, and ◦ denotes the Hadamard product.

### 1.4 The predictive factorization algorithm

Current factorization methods are not predictive and can only be used for signal inference. In the case of NMF methods [5], this arises from the gradient descent methods used for optimization. Often, an alternating gradient descent is performed, where one matrix is kept fixed while the other is optimized. The drawback for a predictive approach based on *A *is that for a given NMF signal matrix *S *the corresponding *A *is not uniquely defined.

For any predictive approach, training a model requires that *A *is treated as a function of *S *and *X*. This, to our best knowledge, is not the case in current factorization approaches.

Here, we solve this problem by using the Moore-Penrose Pseudoinverse (MP) of *S *during training to compute *A*. The MP is uniquely defined for any matrix *S*. Let *S*^+ ^denote the MP of *S *being defined by the following properties

(7) $$SS^{+}S=S,S^{+}SS^{+}=S^{+},{\left(SS^{+}\right)}^{T}=SS^{+},{\left(S^{+}S\right)}^{T}=S^{+}S$$

Using these rules, it is easy to show that

(8) $$X=AS\Leftrightarrow XS^{+}=A$$

using (7) and assuming the existence of the quadratic matrix (*SS*^+^)^-1^. Now, *A *is clearly defined as a function of *X *and *S *and we have solved the problem of the uniqueness.

Used in the following sections, we derive the differential for *S*^+^. Therefore, we adopt the notation from [13] to compute *dS*^+ ^as

$$\begin{array}{lll} \;\;\;\;\;dS & = & d\left(SS^{+}S\right)=\left(dS\right)\left(S^{+}S\right)+S\left(dS^{+}\right)S+\left(SS^{+}\right)\left(dS\right)\\ \Leftrightarrow S\left(dS^{+}\right)S & = & dS-\left(dS\right)\left(S^{+}S\right)-\left(SS^{+}\right)\left(dS\right)\\ \;\;\Leftrightarrow dS^{+} & = & {\left(S^{+}S\right)}^{-1}S^{+}\left(dS\right)S^{+}{\left(SS^{+}\right)}^{-1}\\ & & -{\left(S^{+}S\right)}^{-1}S^{+}\left(dS\right)\left(S^{+}S\right)S^{+}{\left(SS^{+}\right)}^{-1}\\ & & -{\left(S^{+}S\right)}^{-1}\left(S^{+}S\right)S^{+}\left(dS\right)S^{+}{\left(SS^{+}\right)}^{-1}\\ \;\;\Leftrightarrow dS^{+} & = & -S^{+}\left(dS\right)S^{+}. \end{array}$$

Together with the two loss functions and the MP differential, all ingredients are available for the *BrierScoreMF *algorithm. First, we join both loss functions into a combined minimization problem

$$\begin{array}{lll} \underset{S,b}{\min\;}ℒ(S,\vec{b}) & = & ℱ{\left(X,XS^{+}S\right)}^{2}+\leftarrow \left(Y,XS^{+}-1{\vec{b}}^{T}\right)\\ & = & \text{tr}{\left(X-XS^{+}S\right)}^{T}\left(X-XS^{+}S\right)\\ & & +\kappa \text{tr}{\left(Y\circ \left(XS^{+}-1{\vec{b}}^{T}\right)-1\right)}^{T}\left(Y\circ \left(XS^{+}-1{\vec{b}}^{T}\right)-1\right) \end{array}$$

and substitute *A *= *XS*^+^. Thus, the complete loss function is easily expressed using matrix terms, where we have omitted the sizes of the **1**-matrices for simplicity. Furthermore, we have used that is suffices to optimize a monotonic transformation of *F *[[13], p. 129 Theorem 9].

To find a minimizer of the *L*, we compute the differential

$$\begin{array}{lll} dℒ(S,b) & = & d\text{tr}{\left(X-XS^{+}S\right)}^{T}(X-XS^{+}S)\\ & & +\kappa d\text{tr}{\left(Y\circ (XS^{+}-1{\vec{b}}^{T})-1\right)}^{T}(Y\circ (XS^{+}-1{\vec{b}}^{T})-1) \end{array}$$

For the first summand, we compute

$$\begin{array}{l} \;d\text{tr}{\left(X-XS^{+}S\right)}^{T}\left(X-XS^{+}S\right)\\ =2\text{tr}{\left(X-XS^{+}S\right)}^{T}d\left(X-XS^{+}S\right)\\ =-2\text{tr}{\left(X-XS^{+}S\right)}^{T}Xd\left(S^{+}S\right)\\ =-2\text{tr}{\left(X-XS^{+}S\right)}^{T}X\left(\left(dS^{+}\right)S+S^{+}\left(dS\right)\right)\\ =-2\text{tr}{\left(X-XS^{+}S\right)}^{T}X\left(-S^{+}\left(dS\right)S^{+}S+S^{+}\left(dS\right)\right)\\ =2\text{tr}{\left(X-XS^{+}S\right)}^{T}XS^{+}\left(dS\right)S^{+}S-2\text{tr}{\left(X-XS^{+}S\right)}^{T}XS^{+}\left(dS\right) \end{array}$$

Using tr*A^T ^*(*B *◦ *C*) = tr(*A^T ^*◦ *B^T^*) *C *[[13]13, p. 45, Theorem 7 (a)], the second summand derives to

$$\begin{array}{l} \;d\text{tr}{\left(Y\circ \left(XS^{+}-1{\vec{b}}^{T}\right)-1\right)}^{T}\left(Y\circ \left(XS^{+}-1{\vec{b}}^{T}\right)-1\right)\\ =2\text{tr}{\left(Y\circ \left(XS^{+}-1{\vec{b}}^{T}\right)-1\right)}^{T}d\left(Y\circ \left(XS^{+}-1{\vec{b}}^{T}\right)-1\right)\\ =2\text{tr}{\left(Y\circ (XS^{+}-1{\vec{b}}^{T})-1\right)}^{T}\left(Y\circ \left(dXS^{+}\right)\right)\\ \;\;-2\text{tr}{\left(Y\circ \left(XS^{+}-1{\vec{b}}^{T}\right)-1\right)}^{T}\left(Y\circ 1{\left(d\vec{b}\right)}^{T}\right)\\ =-2\text{tr}{\left(\left(Y\circ \left(XS^{+}-1{\vec{b}}^{T}\right)-1\right)\circ Y\right)}^{T}XS^{+}\left(dS\right)S^{+}\\ \;\;-2\text{tr}{\left((Y\circ \left(XS^{+}-1{\vec{b}}^{T}\right)-1\right)\circ Y)}^{T}1{\left(d\vec{b}\right)}^{T} \end{array}$$

Now, consider the term

$$\begin{array}{l} \;{\left(\left(Y\circ \left(XS^{+}-1{\vec{b}}^{T}\right)-1\right)\circ Y\right)}^{T}\\ ={\left(Y\circ \left(XS^{+}-1{\vec{b}}^{T}\right)\circ Y-1\circ Y\right)}^{T}\\ ={\left(XS^{+}-1{\vec{b}}^{T}-Y\right)}^{T}, \end{array}$$

because *Y *◦ *Y *= **1 **and **1 **◦ *Y = Y*. Setting the differential to zero and using the computation rules for the trace, especially tr*ABC *= tr*CAB*, we derive

$$\begin{array}{lll} dℒ(S,b) & = & 2\text{tr}{\left(X-XS^{+}S\right)}^{T}XS^{+}(dS)S^{+}S\\ & & -2\text{tr}{\left(X-XS^{+}S\right)}^{T}XS^{+}(dS)\\ & & -2\kappa \text{tr}{\left(XS^{+}-1{\vec{b}}^{T}-Y\right)}^{T}XS^{+}(dS)S^{+}\\ & & -2\kappa \text{tr}{\left(XS^{+}-1{\vec{b}}^{T}-Y\right)}^{T}1{\left(d\vec{b}\right)}^{T}\\ & = & \text{tr}[(2S^{+}S{\left(X-XS^{+}S\right)}^{T}-2{\left(X-XS^{+}S\right)}^{T}\\ & & -2\kappa S^{+}{\left(XS^{+}-1{\vec{b}}^{T}-Y\right)}^{T})XS^{+}(dS)]\\ & & -2\kappa \text{tr}{\left(XS^{+}-1{\vec{b}}^{T}-Y\right)}^{T}1{\left(d\vec{b}\right)}^{T}\\ & = & 0. \end{array}$$

As both tr terms relate to distinct differentials, we first obtain that

$$0=XS^{+}-1{\vec{b}}^{T}-Y\Leftrightarrow XS^{+}=Y+1{\vec{b}}^{T}\equiv W$$

for the coefficients of $d\vec{b}$.

Assuming *XS^+ ^*≠ **0 **and substitution of *W *back into $0=dℒ(S,\vec{b})$ yields

(9) $$\begin{array}{lll} \;\;0 & = & 2S^{+}S{\left(X-WS\right)}^{T}-2{\left(X-WS\right)}^{T}-2\kappa S^{+}{\left(W-1b^{T}-Y\right)}^{T}\\ & = & 2\left(S^{+}S-E\right)\left(X-WS\right)-2\kappa 0\\ \Rightarrow X & = & WS\\ \Leftrightarrow S & = & W^{+}X={\left(Y+1{\vec{b}}^{T}\right)}^{+}X \end{array}$$

To this end, we have found a solution for the predictive matrix factorization problem using the Brier Score as classification loss and the Frobenius norm as factorization loss. Moreover, the solution is fully determined by a single *k *× 1 vector $\vec{b}$ that allows the computation of the factorized signal matrix *S *as well as the computation of the predictive coefficient matrix

$$A=X^{*}S^{+}=X^{*}{\left({\left(Y+1{\vec{b}}^{T}\right)}^{+}X\right)}^{+}$$

for unknown data X*.

Now, the final problem of finding the vector $\vec{b}$ remains. In the present approach, we found that optimizing the following target function yields best performance

$$O(\vec{b})\equiv \frac{1}{r}\prod_{i}\left(s_{i}t_{i}\right)$$

where *s*_i _and *t_i _*denote the cross-validated sensitivities and specificities, and *r *denotes the cross-validated reconstruction error. Using numerically computed gradients for $\vec{b}$ in combination with a BFGS local search method [14] to optimize O completes the *BrierScoreMF*.

We conclude this section with an interpretation of equation (9). First, we note that the *BrierScoreMF *has very few parameters, namely $\vec{b}\in {\mathbb{R}}^{k}$, which minimizes the probability of over-fitting (Occam's Razor), but also hampers the algorithm in obtaining high prediction performance. Next, the computation of *S *involves both, the design matrix *X *used for training and the class matrix *Y *. Thus, using the known classes and a linear offset $\vec{b}$ the training data is projected by the MP of ${\left(Y+1{\vec{b}}^{T}\right)}^{+}$ to a transformed matrix *S*.

Consequently, the training information *Y *and *X *are compressed together with the learned variables $\vec{b}$ in *S*. In this way, our new factorization method is similar to nearest neighbor classifiers, which also store the training data itself while learning a threshold value for classification.

All software used in this article is freely available from the author.

## Results and Discussion

This section empirically compares the performance of the *BrierScoreMF *with linear Support Vector Machines (SVM) [15]. Therefore, we sample synthetic signal functions together with class and coefficient matrices for training both machine learning models. This setting was specifically designed with regard to the application case of IR spectroscopy. Finally, we train both algorithms on a real world IR data set comprising various diseases for classification.

We would like to note in advance that this comparison is not totally fair. SVM are pure classification algorithms that are statistically highly robust and achieve very high performance. In contrast, the *BrierScoreMF *is a factorization method designed for both, signal decomposition and prediction. Therefore, the problem solved by our algorithm is more constrained than the SVM.

In addition, our method has less degrees of freedom. To infer a *BrierScoreMF *model only *k*, being the number of classes, variables are optimized. Contrarily, even a linear SVM has *m*, being the number of input dimensions, variables to specify a predictive model. In our case, *m *= 3200 and *k *= 16, thus rendering *BrierScoreMF *the less flexible model. In addition, our method is a native multi-class algorithm where one model suffices to explain all classes. In contrast, the employed multi-class linear SVM are trained in one-versus-one mode resulting in 16 · 15 = 240 models used for prediction. In terms of Occam's razor, our model is the more simple method with an generative model suitable for prediction.

Thus, we compare both algorithms for baseline reasons and not to demonstrate the superiority of the *BrierScoreMF*. A comparison to actual factorization methods is planned as future work, because the question for fair performance measures for this task turns out to be far more delicate.

### 1.5 Experiments on synthetic data sets

IR spectra of chemical compounds and mixtures are smooth functions of the wavelength. In general, the measurement ranges from 400*cm*^ -1 ^to 4000*cm*^ -1 ^for Fourier-Transform Infrared Spectroscopy. However, we have chosen to sample base signals from Sobolev Spaces [16] defined on the range 0[1] as smoothness is more important than the signal domain.

Sobolev spaces are function spaces defining smooth functions. In a Sobolev space, smoothing a function means shrinking higher order coefficients towards zero. Therefore, sampling signals from this family of functions yields appropriate spectra that are smooth. We chose the Fourier basis *ϕ_i_*(*x*)

$$\varphi_{1}(x)=1,\;\varphi_{2j}(x)=\frac{1}{\sqrt{2}}\cos(2j\pi x),\;\varphi_{2j+1}(x)=\frac{1}{\sqrt{2}}\sin(2j\pi x),\;j=1,2,\;\ldots$$

from which signals

$$f(x)=\sum_{j=1}^{\infty }\theta_{j}\varphi_{j}(x).$$

where sampled by their coefficients *θ*_*j*_.

In this experiment, each synthetic data set is defined by four parameters: a seed for the random number generator to make the experiment reproducible, the number *n *of samples generated for the data set, the number *k *of classes contained in the data set, and the number *m *of feature dimensions. We used 5-fold cross-validation (CV) to estimate the prediction performance in terms of sensitivity *s_i _*and specificity *t_i _*as well as the reconstruction error *r*

$$s_{i}\equiv \frac{TP_{i}}{TP_{i}+FN_{i}},\;t_{i}\equiv \frac{TN_{i}}{TN_{i}+FP_{i}},\;r\equiv ∥X-XS^{+}S{∥}_{F}$$

where *TP_i _*denotes the true positives, *TN_i _*the true negatives, *FP_i _*the false positives, and *FN_i _*the false negatives of class *c_i_*. Note that the *BrierScoreMF *employs an inner cross-validation loop for performance estimation, therefore the outer cross-validation measures the true generalization error of our model.

The generation of a data set was performed as follows: First, the seed of the random number generator was set. Then, the $\vec{b}$ vector was sampled from a uniform distribution. After that an *n*-array *y *of classes was obtained by sampling classes with replacement from *c_1_*, . . ., *c_k_*. This was followed by sampling the order *o *of the Sobolev space by drawing an integer out of the range [1, 100]. Based on this, a matrix *T *containing *o *signal coefficients for each of the *k *signals was drawn from a uniform distribution. Finishing the sampling round, we finally drew the coefficient matrix *A *from a uniform distribution.

First, the matrix *S *containing the *m *measurements at equally spaced coordinates between 0[1] was computed from the coefficient matrix *T *(*d *= 3200). Then, the class matrix *Y *was constructed from the class array *y *by setting appropriate entries on +1 and every other entry to -1. Finally, we processed the coefficient matrix to relate to *Y *as follows: Each entry of *A *was scaled to the range [0, *b_i_*) for negative corresponding entries in *Y *and transformed into the range [*b_i_*, 1] for positive ones. After that all entries relating to negative *Y *-entries were scaled such that $\sum_{j}a_{ij}=1\forall i$. Given *A *and *S *we finally computed *X *= *AS*, completing the synthetic data set.

**Figure 2:**
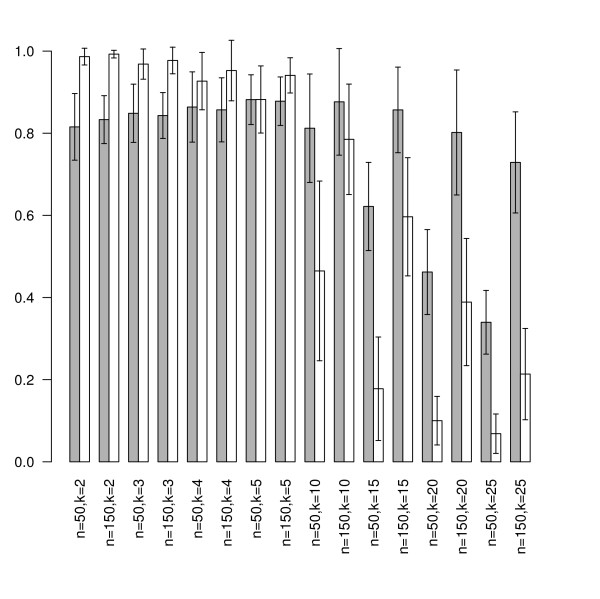
**Sensitivity performance on synthetic data**. This figure shows the achieved sensitivities of *BrierScoreMF *(gray) vs linear SVM (white) on the synthetic data sets for *m *= 50 and *n *∈ {50,100} and a varying number of classes *k *∈ {2, 3, 4, 5, 10, 15, 20, 25}. For low k values, the SVM is better than the BrierScoreMF algorithm. However, for more than 10 classes, *BrierScoreMF *clearly outperforms multi-class linear SVM. These results were obtained by averaging 100 seeded comparisons.

**Figure 3:**
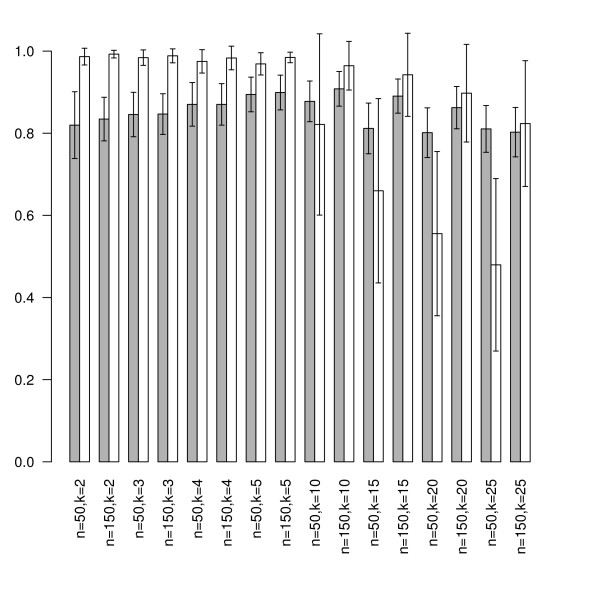
**Specificity performance on synthetic data**. This figure shows the achieved specificities of *BrierScoreMF *(gray) vs linear SVM (white) on the synthetic data sets for *m *= 50, n ∈ {50, 150} and a varying number of classes *k *∈ {2, 3, 4, 5, 10, 15, 20, 25}. For low k values, the SVM outperforms the BrierScoreMF algorithm. However, for more than 10 classes, the specificity of the linear SVM deteriorates, while BrierScoreMF achieves approximate constant prediction performance. These results were obtained by averaging 100 seeded comparisons.

In this way, we obtained 4800 synthetic data sets using 100 seeds for each combination of *n *∈ {50, 100, 150}, *k *∈ {2, 3, 4, 5, 10, 15, 20, 25} and *m *∈ {50,100}. On each data set, first the *BrierScoreMF *and subsequently the linear multi-class SVM from the R package e1071 was trained [17]. Thus, a direct performance comparison based on 5-fold CV was obtained. The prediction results are shown in Figures 2 and 3.

First, we found that there exist no significant differences in the performance behavior with respect to the input dimensions *m *for both algorithms. Inspection of the class parameter reveals that the linear SVM is superior to the *BrierScoreMF *for problems with less than five classes. Nonetheless, in these categories the *BrierScoreMF *achieves sensitivities and specificities around 0.8 with a standard deviation of less than 0.1. For problems with the number of classes between 10 and 25 the *BrierScoreMF *achieves superior sensitivities and *specificities *to the linear SVM. However, if the number of training samples is large (*n *= 150), the linear SVM obtains competitive specificities again. In summary, we find that the prediction performance of the *BrierScoreMF *decreases slower than the performance of the SVM with increasing class size. Finally, we note that in contrast to the SVM the standard deviations of the *BrierScoreMF *for sensitivity do not exceed 0.15 and for specificity 0.08. In conclusion, we have characterized and compared the prediction performance of the *BrierScoreMF *on synthetic data with a state of the art machine learning method. As explained, the *BrierScoreMF *solves a more complex system by generating an interpretable signal factorization, which balances the performance loss.

In the next section, we present results of the *BrierScoreMF *obtained by training on real IR spectra.

### 1.6 Experiments on a clinical data set

**Figure 4:**
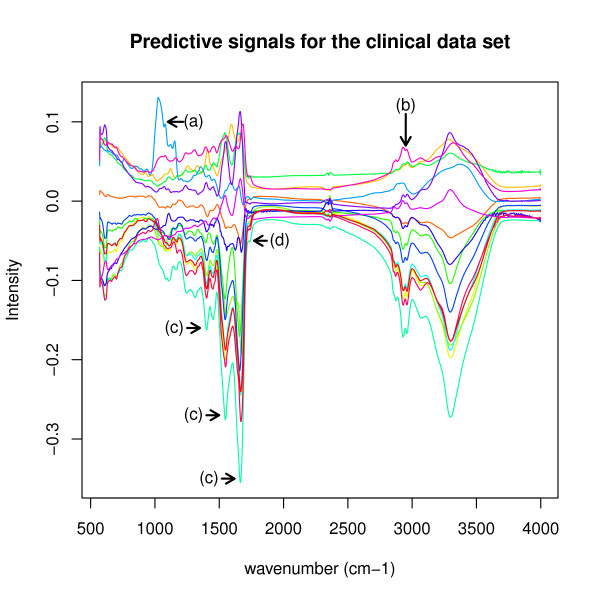
**Predictive signals for the clinical data set**. This figure shows the 16 predictive signals inferred for the clinical data set. Four examples for interesting peaks are marked by (a) to (d) and are discussed in the text.

**Figure 5:**
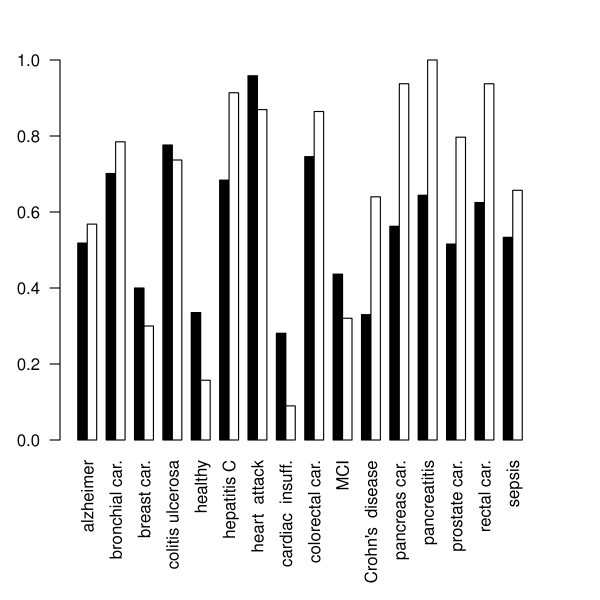
**Sensitivity performance on clinical data**. This figure shows the result of the sensitivity evaluated by 5-fold CV of *BrierScoreMF *vs linear SVM on the clinical data set. Here, we have abbreviated "Cancer" by "car." and "Mild Cognitive Impairment" by "MCI". The dark bars denote the *BrierScoreMF*, while white bars refer to the linear SVM.

**Figure 6:**
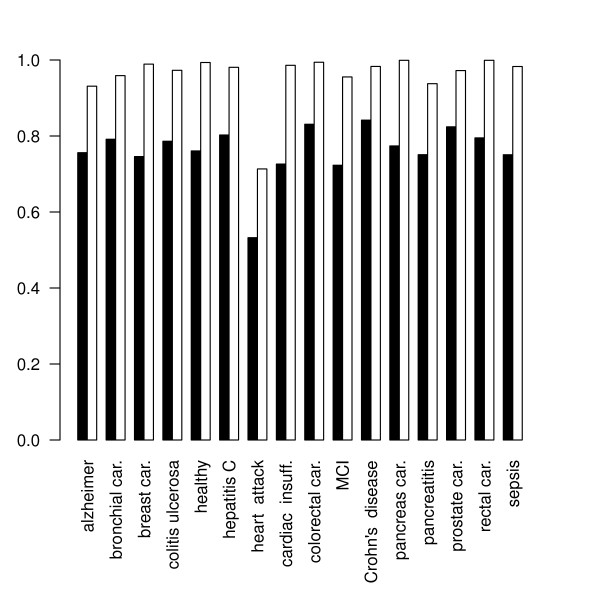
**Specificity performance on clinical data**. 5-fold CV specificity of *BrierScoreMF *vs linear SVM on the clinical data set. Here, we have abbreviated "Cancer" by "car." and "Mild Cognitive Impairment" by "MCI". The dark bars denote the *BrierScoreMF*, while white bars refer to the linear SVM.

Next, we applied the *BrierScoreMF *to real world data. Therefore, we have reused the IR spectra of blood serum measured for the study in [18]. Therein, serum samples were collected at the University Hospital Heidelberg, the University Hospital Mannheim, and the St. Vencentius Krankenhaus in Karlsruhe, while the healthy control was obtained from the blood donating center in Mannheim. In total, 15 different diseases were collected and analyzed. However, reference [18] combines the classes MCI and Alzheimer, Colitis Ulcerosa and Morbus Crohn, heart insufficiency and heart infarction, as well as colorectal carcinoma and rectal carcinoma and, therefore, reports 12 diseases. In this work, we predict the more detailed classifications. For each IR spectrum, 1 μ*l *of serum was diluted to 3 μ*l *of distilled water, placed and dried on a 384 well Si-sample carrier plate. Then, the plate was measured on a Bruker Tensor 37 Fourier Transform IR spectrometer (Bruker Optics GmbH Ettlingen, Germany). In total, each sample was measured at least at three different days having randomized positions on the 384 well plate to avoid environmental effects. In this work, subsequent data processing consisted of the removal of all triplicates having a pairwise Pearson correlation of less than 0.95. All remaining triplicates were averaged before Savitzky-Golay smoothing (filter length was set to 15). Finally, we employed 5-fold CV to estimate the prediction performance of both SVM and *BrierScoreMF*. The results of this evaluation are shown in Figures 4, 5, and 6.

We found that the linear SVM was often superior to the *BrierScoreMF*. It was highly specific (Figure 6) while being less sensitive (Figure 5) than our method in some cases. As explained above, this outcome was expected as the linear SVM has more degrees of freedom (*m *= 3200) compared to *BrierScoreMF *(*k *= 16). In addition, training one-versus-one classifiers provides additional robustness with respect to noise as the classification problem is separated into smaller pieces. Whereas our algorithm is a native multi-class algorithm that is additionally constrained to yield an interpretable factorization.

However, our method achieved an estimated reconstruction error of 1.5325 × 10^-04 ^per matrix entry for this data set. The sensitivity ranges from 0.2809 to 0.9586, while specificity ranges from 0.5324 to 0.8417. In addition, it infers interpretable and predictive signals that may lead to further insight into characteristic disease signals, Figure 4.

**Figure 7:**
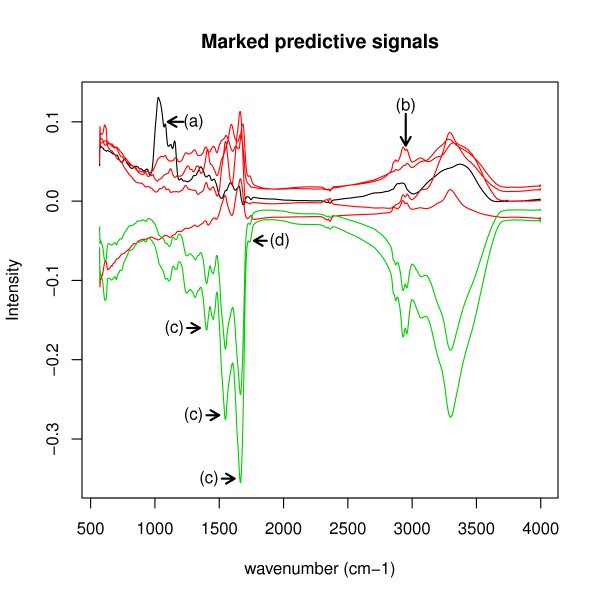
**Marked predictive signals**. This figure shows the marked signals. Black denotes the signal for colorectal carcinoma. Red denotes the signals for bronchial carcinoma, pancreas carcinoma, prostate cancer, and pancreatitis. Greens denotes the signals for heart attack and heart insufficiency. The heart signals (green) are correlated with a Pearson correlation of *ρ *= 0.9981. Here, the heart attack signal shows a higher amplitude than the heart insufficiency signal, which may be a result of the increased troponin T or troponin I levels during heart attack compared to the slightly increased levels within heart insufficiency.

**Figure 8:**
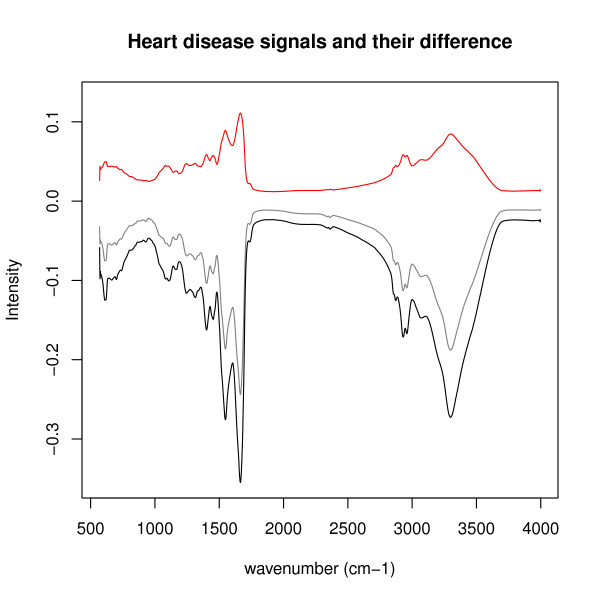
**Heart disease signals and their difference**. This figure plots the heart disease signals for heart attack (black) and heart insufficiency (gray) and their absolute signal difference (red). The black signal is a scaled version of the gray signals, which can be explained by the increased of troponin I and troponin I levels of heart attack patients compared to heart insufficiency patients. Both signals reveal a Pearson correlation of *ρ *= 0.9981, which was the highest correlation among the inferred signals.

To demonstrate the ability of the *BrierScoreMF *to discover interesting signal features in IR spectra, we now focus on four exemplary signal peaks in Figure 4, named (a), (b), (c) and (d). Figure 7 shows only the discussed signals. The peak at (a) belongs to the colorectal carcinoma signal. It is within 1100-1150*cm*^-1 ^and, therefore, may belong to the region were normally the DNA/RNA ribose CO stretching vibrations appear [1]. In colorectal carcinoma, this potentially indicates an increase of DNA/RNA damage by post-transcriptionally modified nucleic acids induced by cancer progression [19]. The peak at (b) is at 2700-2950*cm*^-1 ^and, thus, is in the region of the CH-group of phospholipids. Signals comprising peaks at (b) include bronchial carcinoma, colorectal carcinoma, pancreas carcinoma, pancreatitis, as well as the prostate carcinoma. Here, the phospholipid groups may relate to inflammatory signals in the blood responding to cancer [20]. The signal peaks marked with (c) relate to amid groups, while (d) indicates an ester of phospholipids. The disease specific signal showing these peaks (c) and (d) belong to heart attack (Figure 8). It is known that lipids form plugs that are a major cause for heart attacks, which could correlate the signals at (d) [21]. Additionally, we measured the Pearson correlation between the heart attack and the heart insufficiency signal resulting in *ρ *= 0.9981, which equals the maximum positive correlation within the inferred signals. Consequently, our algorithm was able to detect several interesting disease specific signals for further research.

The additional files provide supplementary results for training without the water peaks (Additional file 1)) as well as the detailed prediction performance of the *BrierScoreMF *method on the clinical dataset (Additional file 2).

## Conclusions

In this work, we have presented the *BrierScoreMF *algorithm for factorization of additive signals. The ultimate goal was to employ IR spectra obtained from blood samples to classify patients based on disease specific signals. We have established a performance baseline for our method on both, synthetic and real world data. Yielding interpretable base signals, our factorization obtains comparable prediction performance on synthetic data sets comprising more than 10 classes. On real world data, we measure sensitivities as well as specificities of up to 0.8.

Our factorization method combines both tasks of prediction and signal inference. Therefore, we are confident that our work constitutes the basis for further development of similar factorization algorithms. Future research should focus on improving the prediction performance of *BrierScoreMF*, as well as on a correct comparison with actual factorization methods. Also, the integration of non-negativity constraints into our algorithm is of practical interest.

## Authors' contributions

CH developed the algorithm and designed the experiment. PL also contributed to the experimental design. ED and JB collected and measured the clinical IR spectra. BK and AZ supervised the project. All authors read and drafted the manuscript.

## Supplementary Material

Additional file 1 **Comparison of the BrierScoreMF performance excluding Water Absorbtion Peaks**. Here, we compare the BrierScoreMF performance on the clinical dataset when training with and without the water absorption peaks located at [2200-2270 1/cm] and [3200-3700 1/cm]. We find that omitting these regions does not significantly alter the prediction performance. This file can be opened with Microsoft Word 2002, Open Office Writer 3.1.1, or similar word processor programs. Click here for file

Additional file 2 **Detailed BierScoreMF performance**. Here, we provide the BierScoreMF performance on the clinical dataset in terms of True Positives, True Negatives, False Positives, False Negatives, as well as Sensitivity, Specificity, and Matthews Correlation Coefficient. This file can be opened with Microsoft Excel 2002, Open Office Calc 3.1.1, or similar spreadsheet applications. Click here for file
