# Supplementary material for: A factorization method for the classification of infrared spectra
Source: BMC Bioinformatics. 2010 Nov 15;11:561. doi: 10.1186/1471-2105-11-561 (PMC3247165; doi:10.1186/1471-2105-11-561)
Supplement: Additional file 1 — Comparison of the BrierScoreMF performance excluding Water Absorbtion Peaks. Here, we compare the BrierScoreMF performance on the clinical dataset when training with and without the water absorption peaks located at [2200-2270 1/cm] and [3200-3700 1/cm]. We find that omitting these regions does not significantly alter the prediction performance. This file can be opened with Microsoft Word 2002, Open Office Writer 3.1.1, or similar word processor programs. [file 1471-2105-11-561-S1.DOC]

# Evaluation of the impact of the water peaks on the prediction performance of the BrierScoreMF algorithm

Carsten Henneges, Pavel Laskov, Endang Darmawan, Jürgen Backhaus,
Bernd Kammerer, Andreas Zell

Here, we assess the impact of water peaks on the prediction performance of the BrierScoreMF algorithm. Therefore, we first exclude the spectral ranges between [2200, 2500] and
[3200, 3700], where water absorbtions mainly occur due to 0-H stretching and O-H deformation oscillations. Subsequently, we trained the BrierScoreMF algorithm on the water-reduced spectral dataset and measure the class prediction performance in terms on sensitivity and specificity.

The following graphics compare the sensitivities and specificities of Figures 5 and 6 in the Manuscript to those obtained by training without the water peaks. Figure 1 shows the distribution of the differences in sensitivity (red) and specificity (green)

$$\begin{matrix} \text{Diff}_{\text{Sensitivity}}\text{ = } \text{Sensitivity}_{\text{Water}}-\text{Sensitivity}_{\text{No Water}} \\ \text{Diff}_{\text{Specificity}}\text{= Specificity}_{\text{Water}}-\text{Specificity}_{\text{No Water}} \end{matrix}$$

between the two runs. We find that both distributions are closely centered near zero. The differences in specificities range from -0.1 to 0.1 and reveal a narrow peak. The differences in sensitivity has a broader distribution ranging from -0.2 to 0.1, but is centered at 0.05. However, both distributions are centered around zero, which indicates a zero to small impact on the prediction performance. Therefore, we leave the impact of water adjustment for factorisation methods to subsequent studies.

Nonetheless, we provide a direct comparison of the BrierScoreMF performance in Figure 2 and Figure 3.


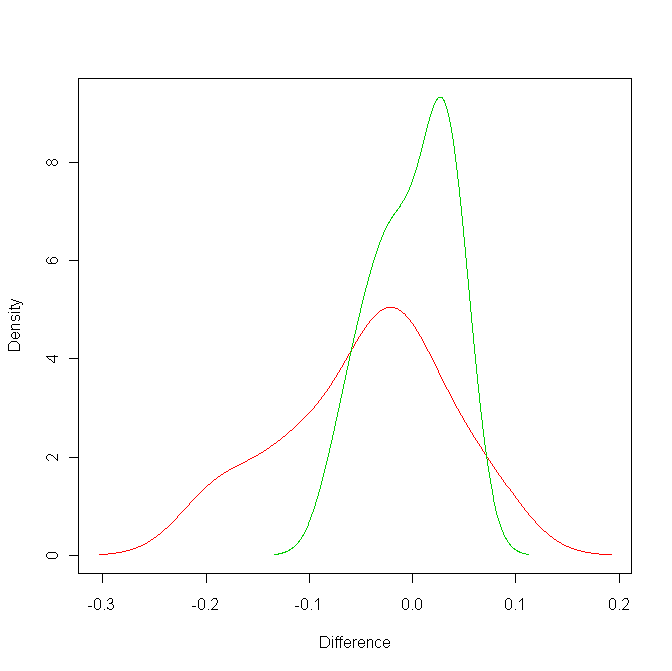


Figure 1 Difference distributions of sensitivity (red) and specificity (green).


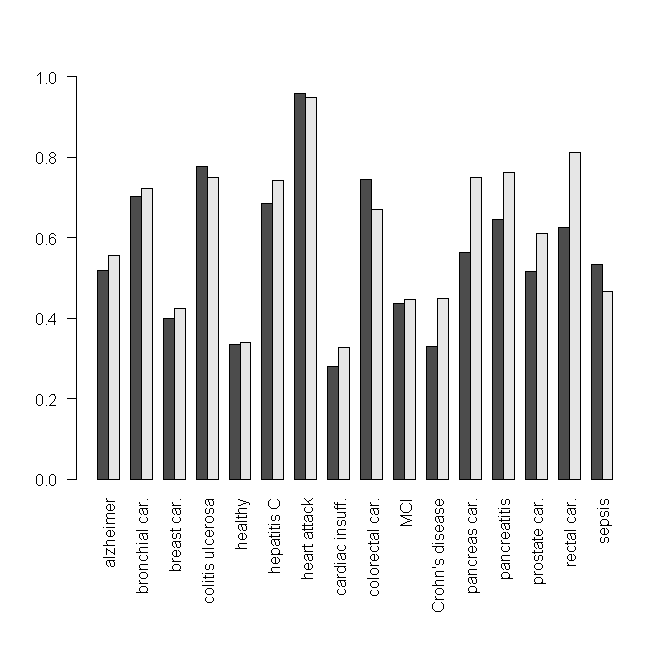


Figure 2 Sensitivity of BrierScoreMF training with water peaks (black) and without water peaks (gray).


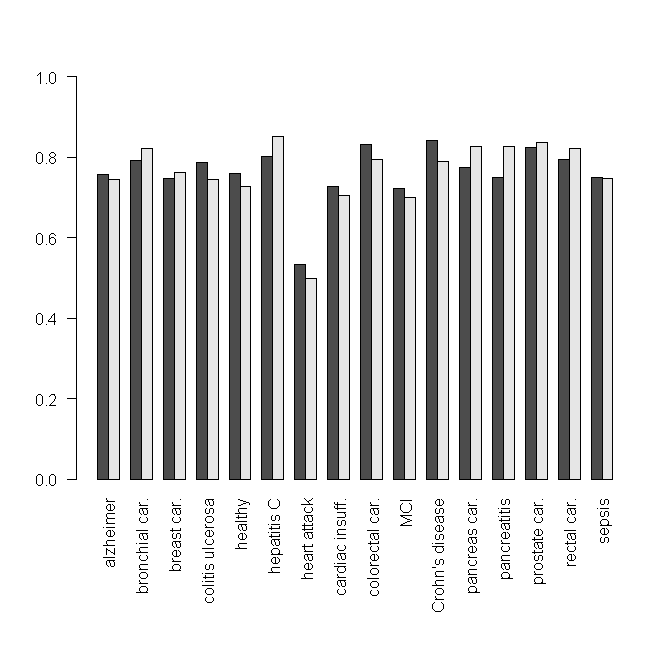


Figure 3 Specificity of BrierScoreMF training with water peaks (black) and without water peaks (gray).
